# Supplementary material for: Structure Identification of Two Polysaccharides from Morchella sextelata with Antioxidant Activity
Source: Foods. 2022 Mar 28;11(7):982. doi: 10.3390/foods11070982 (PMC8997402; doi:10.3390/foods11070982)
Supplement: Supplementary file 1 [file foods-11-00982-s001.zip › foods-1651740-supplementary.pdf]

Supplementary material

Methods

1. Antioxidant activity in vitro

DPPH free radical scavenging ability was measured as follows. A sample solution (2 mL) with a concentration of 0.5, 1.0, 1.5, 2.0, and 4.0 mg/mL, respectively was mixed with 2.0 mL of DPPH solution (0.2 mM, formulated with absolute methanol) in a test tube. The final solutions were protected against the light for 30 min. The absolute methanol (2 mL) was used as a blank, and its absorbance was measured at 517 nm. The DPPH free radical scavenging activity rate (%) was calculated using the following equation: Scavenging activity rate (%) = $[A_0-(A_1-A_2)]/A_0 \times 100\%$. where A_0, A_1, and A_2 represent the absorbance of pure DPPH solution, the mixture solution of the sample and DPPH, and the pure sample solution, respectively.

To test ferric reducing ability, polysaccharide solutions of various concentrations (0.5, 1.0, 1.5, 2.0, and 4.0 mg/mL) were prepared and mixed with 2.5 mL potassium phosphate buffer (0.2 M, pH 6.6) and 1 mL potassium ferricyanide (1%, *w*/*v*). The solution was heated at 50 °C for 20 min before centrifuging at 3,000 ×g for 10 min, then added 2.5 mL TFA (10%, *w*/*v*) to the recovered pellet. Subsequently, 0.5 mL ferric chloride (1%, *w*/*v*) and 2.5 mL distilled water were added to the solution, and the reaction continued for 10 min before being measured at 562 nm.

Results and discussion

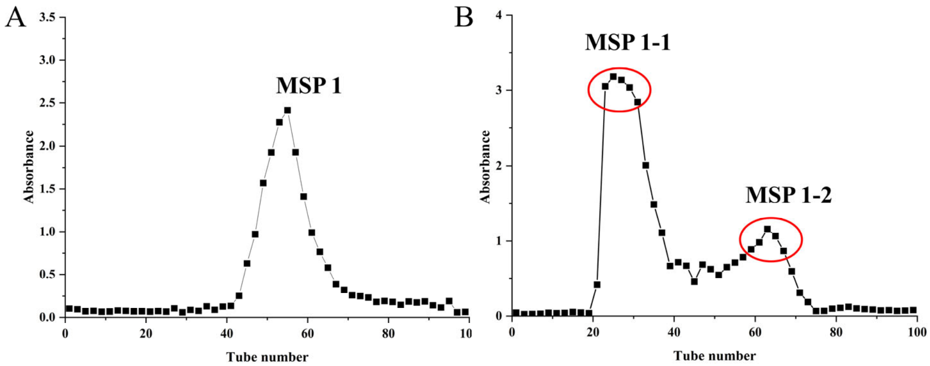

Figure S1. Elution profiles of the *Morchella sextelata* polysaccharides extracted using subcritical water extraction (MSP_{SWE}) on a DEAE-Sepharose fast flow column (A) and Sepharose CL-6B column (B).

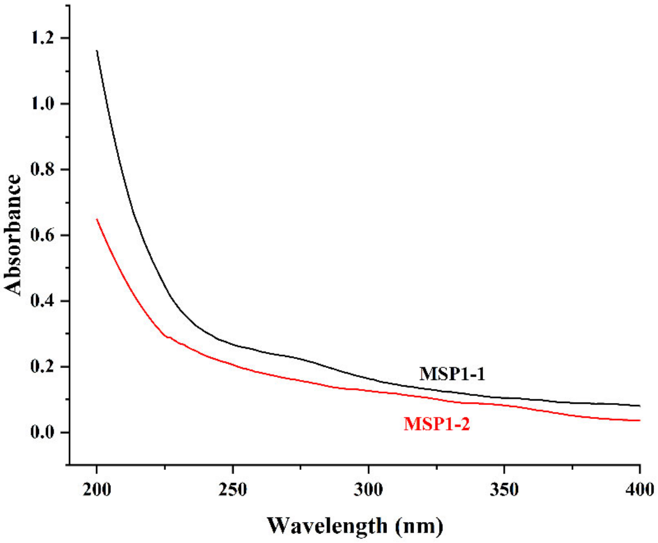

Figure S2. UV spectra of MSP1-1 and MSP1-2.

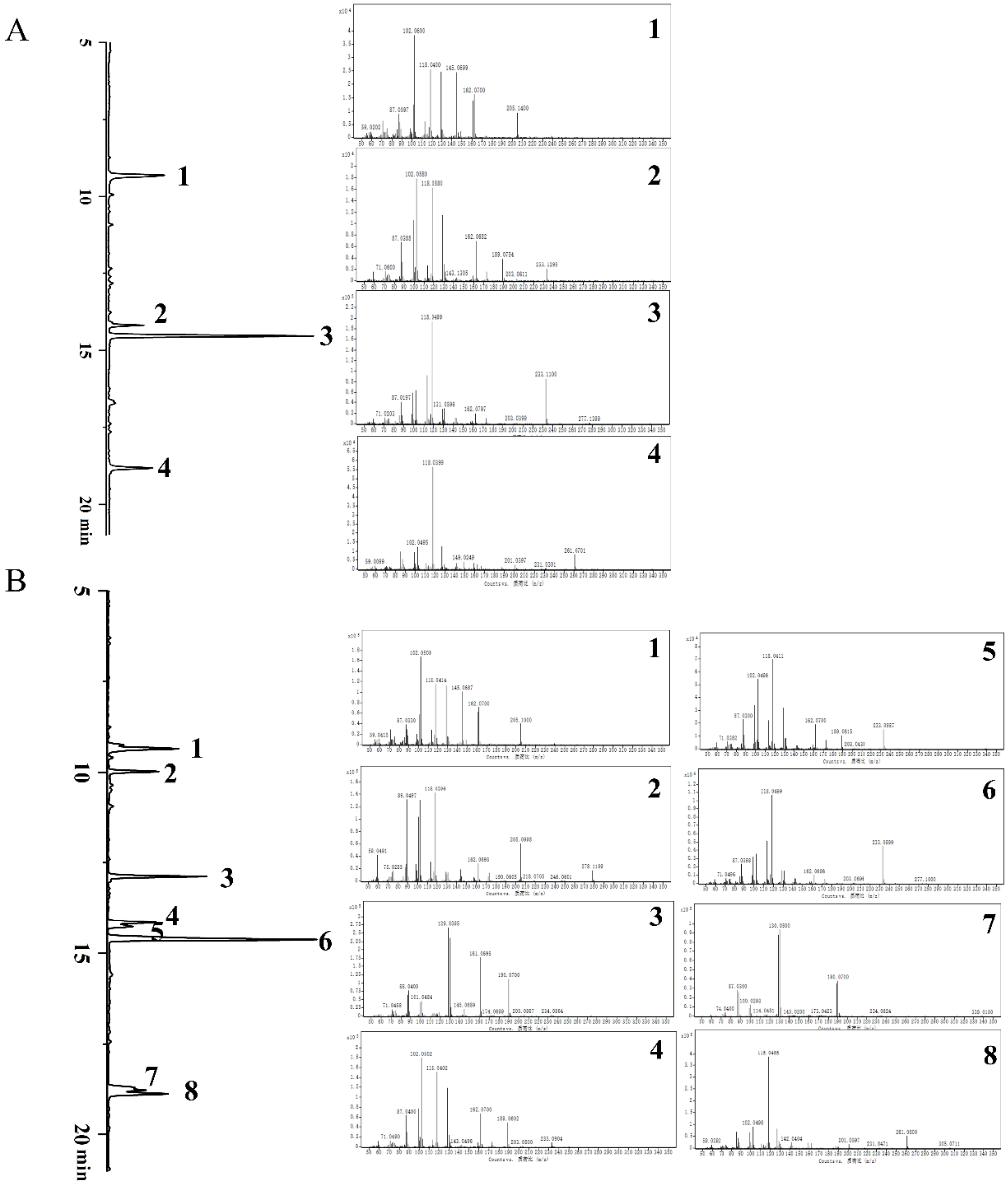

Figure S3. GC–MS profiles of partially methylated alditol acetates of MSP1-1 and MSP1-2. Total ion chromatogram and MS fragments of (A) MSP1-1 and (B) MSP1-2.

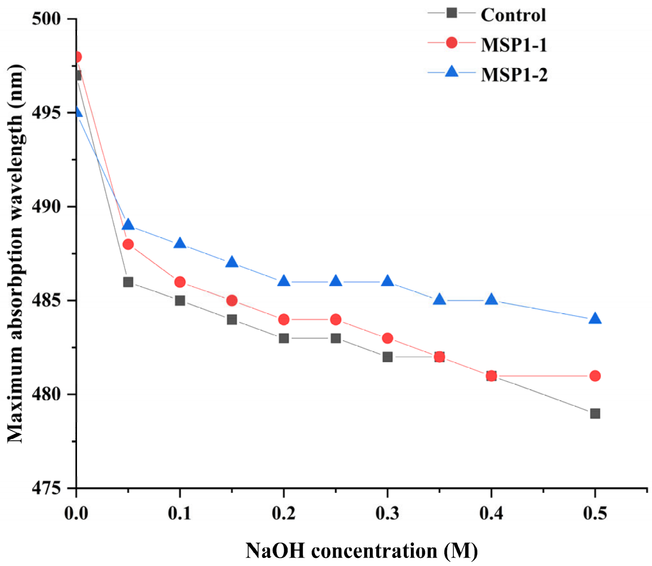

Figure S4. Maximum absorption wavelength when MPS1-1 or MPS1-2 were mixed with Congo red at various NaOH concentrations.

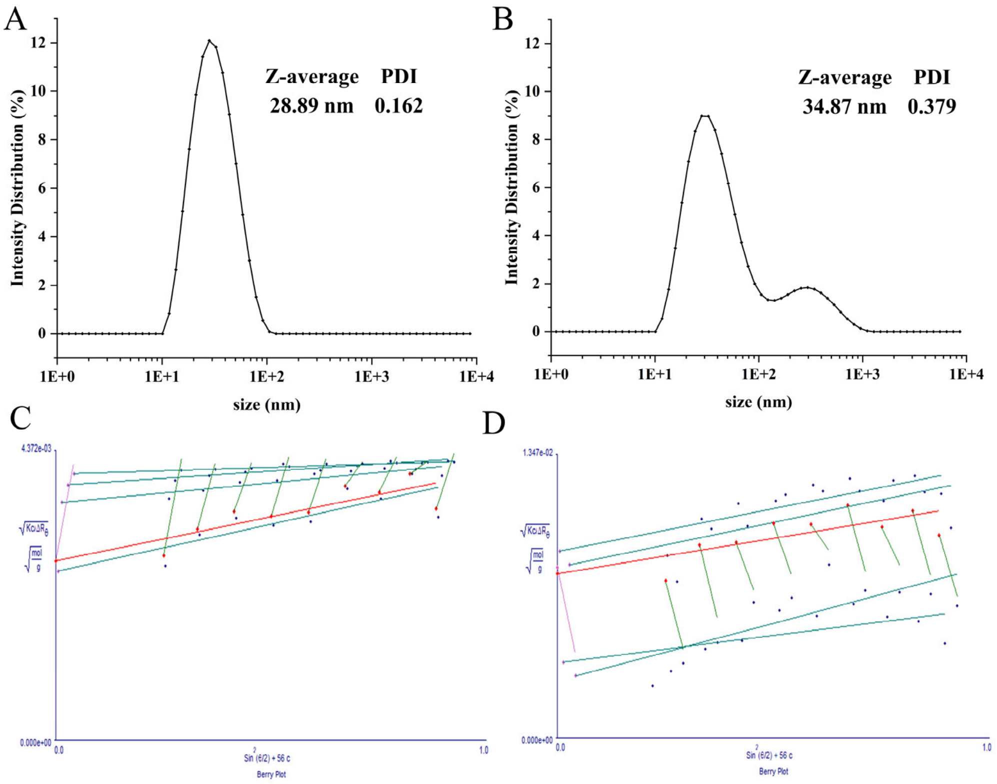

Figure S5. Hydrodynamic radius distributions of MSP1-1 (A) and MSP1-2 (B); Berry plots of MSP1-1(C) and MSP1-2 (D) in 0.9%NaCl solution determined from static light scattering measurements.

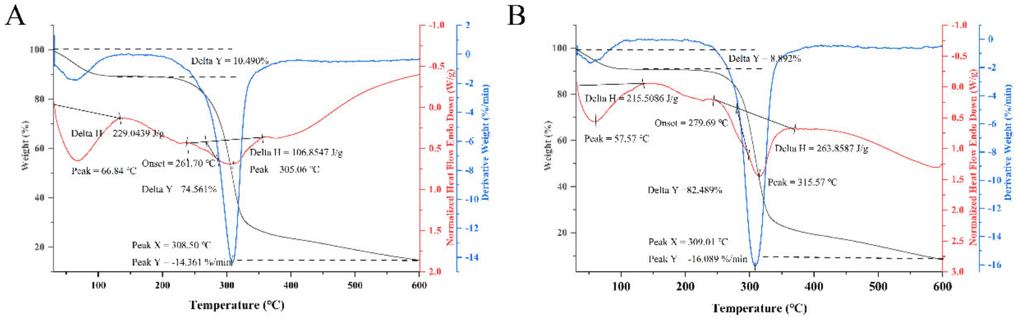

Figure S6. Thermal properties of polysaccharides extracted from *Morchella sextelata*. (A) MSP1-1, and (B) MSP1-2.
